# Corifollitropin alfa or rFSH treatment flexibility options for controlled ovarian stimulation: a *post hoc* analysis of the Engage trial

**DOI:** 10.1186/1477-7827-11-52

**Published:** 2013-06-11

**Authors:** Arthur Leader, Paul Devroey, Han Witjes, Keith Gordon

**Affiliations:** 1 The Ottawa Fertility Centre, Division of Reproductive Medicine, University of Ottawa, Ottawa, ON, Canada; 2 Center for Reproductive Medicine, Universitair Ziekenhuis Brussel, Brussels, Belgium; 3 MSD Oss B.V., Oss, The Netherlands; 4 Merck Sharp & Dohme Corp, Whitehouse Station, NJ, USA

**Keywords:** Protocol flexibility, Controlled ovarian stimulation, Corifollitropin alfa, Recombinant follicle stimulating hormone

## Abstract

**Background:**

We sought to determine the impact of treatment flexibility on clinical outcomes in either a corifollitropin alfa or recombinant follicle-stimulating hormone (rFSH) protocol.

**Methods:**

*Post hoc* analysis of a prospective, multicenter, randomized, double-blind, double-dummy non-inferiority clinical trial (Engage). Efficacy outcomes were assessed on patients from the Engage trial who started treatment on menstrual cycle day 2 versus menstrual cycle day 3, patients who received rFSH step-down or fixed-dose rFSH, patients who received rFSH on the day of human chorionic gonadotropin (hCG) compared with those who did not, and patients who received hCG when the criterion was reached versus those with a 1-day delay.

**Results:**

The effect of each of the treatment flexibility options on ongoing pregnancy rate was not significant. The estimated difference (95% confidence interval) in ongoing pregnancy rate was -4.3% (-9.4%, 0.8%) for patients who started ovarian stimulation on cycle day 2 versus day 3, 1.8% (-4.1%, 7.6%) for patients who received hCG on the day the hCG criterion was met versus 1 day after, 3.2% (-2.1%, 8.6%) for patients who received rFSH on the day of hCG administration versus those who did not, and -5.8% (-13.0%, 1.4%) for patients who received a reduced versus fixed-dose of rFSH from day 8.

**Conclusions:**

Treatment flexibility of ovarian stimulation does not substantially affect the clinical outcome in patients’ treatment following initiation of ovarian stimulation with either corifollitropin alfa or with daily rFSH in a gonadotropin-releasing hormone antagonist protocol.

**Trial registration:**

Trial was registered under ClinicalTrials.gov identifier NCT00696800.

## Background

Corifollitropin alfa, a novel hybrid molecule with sustained follicle-stimulating activity, is a recombinant molecule constructed by coupling the carboxy terminal peptide of the β-subunit of human chorionic gonadotropin (hCG) to the follicle-stimulating hormone (FSH) β-subunit [1]. Due to its pharmacokinetic profile, a single injection of corifollitropin alfa can initiate and sustain multi-follicular growth by maintaining FSH levels above the threshold required for the first 7 days of controlled ovarian stimulation (COS) for *in vitro* fertilization (IVF) and can replace seven daily injections of recombinant FSH (rFSH) [1,2].

The efficacy and safety of corifollitropin alfa have been evaluated in the Engage [3], Ensure [4], and Trust [5] trials. In the Engage trial, ongoing pregnancy rates were assessed in 1506 treated patients after one injection of 150 μg corifollitropin alfa during the first 7 days of stimulation and compared with seven daily injections of 200 IU human rFSH using a standard gonadotropin-releasing hormone (GnRH) antagonist protocol in patients from North America and Europe. Ongoing pregnancy rates of 38.9% for the corifollitropin alfa group and 38.1% for the rFSH group were achieved, with an estimated non-significant difference of 0.9% (95% confidence interval [CI], -3.9% to +5.7%) in favor of corifollitropin alfa [3]. Equivalent ongoing pregnancy rates by treatment were independent of whether patients underwent IVF or intracytoplasmic sperm injection (ICSI), had single or double embryo transfer or embryo transfer on day 3 or day 5. Within each continent there were no differences in the ongoing pregnancy rate and live-birth rate between the two treatment groups; however, between continents, the ongoing pregnancy rate and live-birth rate were considerably higher in North America than in Europe [6].

The new treatment option with corifollitropin alfa in a GnRH antagonist protocol is simpler and more convenient than daily rFSH treatment for patients undergoing assisted reproductive technology (ART) [1,3]. However, there is a perception that there is a loss of flexibility with the corifollitropin alfa treatment option. Flexibility options of clinical importance include: (i) the start day of stimulation, (ii) the option of a 24-h delay in administration of hCG to induce final oocyte maturation, (iii) the option of receiving rFSH on the day of hCG, and (iv) the option of a step-down or fixed-dose of rFSH from day 8. These options were allowed in the Engage trial. In this *post hoc* analysis of the data from the Engage trial we report the impact of treatment flexibility on clinical outcomes in either a corifollitropin alfa or an rFSH regimen.

## Methods

### Study design

The Engage trial was a prospective, multicenter, randomized, double-blind, double-dummy non-inferiority clinical trial. The details of the Engage trial have been published [3] and are summarized below.

The trial was conducted in accordance with principles of Good Clinical Practice and was approved by the appropriate institutional review boards and regulatory agencies. Written informed consent was provided by all subjects.

Patients were treated with either a single subcutaneous (SC) injection of 150 μg (0.5 mL) corifollitropin alfa (N.V. Organon, The Netherlands) or daily 200 IU rFSH (follitropin beta, Follistim AQ Cartridge, N.V. Organon) from day 2 or day 3 of menses (stimulation day 1) for the first 7 days, followed by daily SC injections of rFSH ≤200 IU from stimulation day 8 up to and including the day of hCG administration, depending on the follicular response and at the discretion of the investigator.

Depending on the ovarian response on stimulation day 8, either a maximal daily dose of 200 IU rFSH or a reduced dose (step-down) was allowed, at the discretion of the investigator, in an effort to reduce the risk of severe ovarian hyperstimulation syndrome (OHSS). Similarly, a reduction in the rFSH dose was also allowed from stimulation day 6 onward if too high an ovarian response was noted. At the discretion of the investigator, rFSH administration could be withheld for a maximum of 3 days (coasting) up to and including the day of hCG administration. When no follicles ≥11 mm were visible on ultrasound scan before injection on stimulation day 8, the cycle was cancelled due to insufficient ovarian response.

To prevent premature luteinizing hormone surges, the GnRH antagonist ganirelix 0.25 mg (Ganirelix Acetate Injection, N.V. Organon) was administered once daily SC starting on stimulation day 5 up to and including the day of hCG. Urinary hCG 10,000 IU or 5000 IU (in the case of too high an ovarian response) was administered to induce final oocyte maturation as soon as at least three follicles ≥17 mm were observed by ultrasound scan, or on the next day.

Approximately 34–36 h after hCG injection, oocyte retrieval followed by standard IVF or ICSI was performed. Embryo quality was evaluated for all available embryos on day 3 of culture. Good-quality embryos were those graded as grade 1 (6–10 cells, no fragmentation, and equal blastomere size) or grade 2 (up to 20% fragmentation). To support implantation and early pregnancy, luteal phase support with progesterone (at least 600 mg/d vaginally or at least 50 mg/d intramuscularly [IM]) was started on the day of oocyte retrieval and continued for at least 6 weeks, or either up to menses or a negative pregnancy test performed at least 14 days after embryo transfer.

### Setting

The trial was carried out in 34 different IVF centers, 20 in Europe and 14 in North America.

### Participants

Women aged 18–36 years with a body weight from 60–90 kg, a body mass index of 18–32 kg/m^2^, a menstrual cycle length of 24–35 days and access to ejaculatory sperm and an indication for COS before IVF or ICSI were eligible.

### Variables

Duration of stimulation, number of follicles ≥11 mm on day 8 and on day of hCG, estradiol levels on day 8 and day of hCG, number of oocytes retrieved, number of (good-quality) embryos obtained on day 3, cycle cancellation rate, ongoing pregnancy rate (defined as presence of at least one embryo with heart activity at least 10 weeks after embryo transfer as assessed by ultrasound scan or Doppler, or confirmed by live birth), and incidence of OHSS (data not shown) were assessed.

### Statistical methods

This retrospective analysis compared four patient subgroups: (i) patients who started treatment on menstrual cycle day 2 or day 3, (ii) patients who received hCG on the day that the criterion for administration was reached versus those with a 1-day delay in hCG administration, (iii) patients who did or did not receive rFSH on the day of hCG administration, and (iv) patients who received step-down or fixed-dose rFSH from day 8. These flexibility options were chosen as they are of particular clinical importance [3]. The data used in the current analyses reflect minor corrections to the previously published Engage trial data [3].

The first three subgroups (i-iii) include patients who received hCG and for whom no rFSH dose was withheld for one or more consecutive days including the day of hCG (n = 1437; 716 patients in the corifollitropin alfa group and 721 patients in the rFSH group). This set is referred to as Analysis Set 1. The fourth patient subgroup (iv) included patients who received hCG on or after day 9 and for whom no rFSH dose was withheld for one or more consecutive days from day 8 including the day of hCG (n = 1056; 547 patients in the corifollitropin alfa group and 509 patients in the rFSH group). This set is referred to as Analysis Set 2.

The impact of each of the four treatment options on the ongoing pregnancy rate was assessed using a generalized linear model with binomial distribution, identity link, and covariates. The model based on Analysis Set 1 (Model 1) includes separate factors (covariates) for the three treatment flexibility options (i) cycle day 2, cycle day 3, (ii) no delay, 1-day delay, and (iii) rFSH on day of hCG, no rFSH on day of hCG. The model based on Analysis Set 2 (Model 2) includes a factor for treatment option iv (step-down rFSH, fixed-dose rFSH). Age, treatment group (corifollitropin alfa, rFSH), and region (Europe, North America) were added as covariates to both models. Age is a well-known predictor of pregnancy and region was associated with the chance of ongoing pregnancy in the Engage trial [6]. Based on these models, risk differences after adjusting for covariates were calculated for each of the four treatment options. Interaction terms of treatment group by each treatment flexibility option were added to the models to explore the dependency of the treatment flexibility options on treatment group with respect to their impact on ongoing pregnancy rate.

## Results

### Patient baseline demographics

As might be expected, patient characteristics, including mean age (30.3–31.9 years), weight (68.0–69.5 kg), body mass index (24.7–25.3 kg/m^2^), and duration of infertility (3.0–3.5 years), were similar across all subgroups regardless of treatment flexibility option (those who started treatment on menstrual cycle day 2 or cycle day 3, who received hCG as soon as the criterion was reached or with 1-day delay, who did or did not receive rFSH on day of hCG, and those who received step-down or fixed dose rFSH).

Patients who received a reduced (step-down) rFSH dose had a higher ovarian reserve, as indicated by their baseline antral follicle count (mean [SD], 13.8 [4.1] and 12.9 [3.9] with corifollitropin alfa and rFSH treatment, respectively) than patients who received a fixed 200 IU daily dose of rFSH (11.9 [4.5] and 11.8 [4.3], with corifollitropin alfa and rFSH treatment, respectively). The antral follicle counts were similar in the other treatment flexibility options.

In both treatment groups, a higher percentage of patients with a 1-day hCG delay were from North America (76.6% and 73.3% for corifollitropin alfa and rFSH treatment, respectively) than from Europe (23.4% and 26.7% for corifollitropin alfa and rFSH treatment, respectively). For the other treatment options, the percentage of patients from North America and Europe were similar across the subgroups and varied between 40% and 60%.

### Main results

#### Start day of stimulation on day 2 or 3 of menses

**Figure 1:**
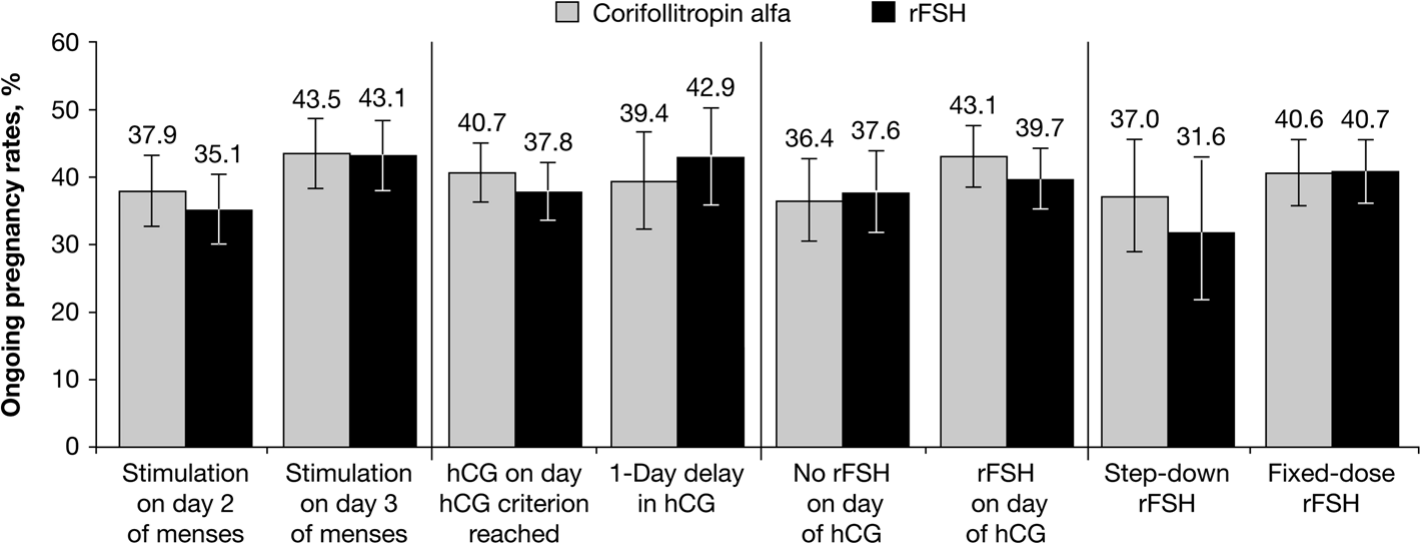
Ongoing pregnancy rates and 95% CIs for the different treatment conditions.

The ovarian response was similar between patients who started treatment on menstrual cycle day 2 and those who started treatment on menstrual cycle day 3 (Table 1). The number of oocytes retrieved in patients who were treated with corifollitropin alfa on menstrual cycle day 2 or day 3 was similar. In the rFSH arm, a similar number of oocytes retrieved was also observed in patients who started treatment on menstrual cycle day 2 or day 3. Cycle cancellation rates were higher in the corifollitropin alfa arm than the rFSH arm. The ongoing pregnancy rates for patients who started treatment on cycle days 2 or 3 were 37.9% and 43.5% in the corifollitropin alfa arm and 35.1% and 43.1% in the rFSH arm, respectively (Figure 1). The estimated difference (95% CI) in ongoing pregnancy rate between cycle day 2 and cycle day 3 adjusted for region, treatment group, age, and treatment flexibility options (ii) and (iii) was -4.3% (-4.3 percentage points) (95% CI: -9.4%, 0.8%) (Table 2). The estimated difference was -6.8% (-11.9%, 1.7%) when adjusted for treatment group only and -4.9% (-10.0%, 0.1%) when adjusted for treatment group, age, and region (data not shown).

**Table 1:** Ovarian response and clinical outcomes: start treatment on cycle day 2 or cycle day 3 (Analysis Set 1)

	**Corifollitropin alfa**		**rFSH**	
	**Day 2**	**Day 3**	**Day 2**	**Day 3**
	**n = 343**	**n = 368**	**n = 353**	**n = 364**
*Duration of stimulation, days, mean (SD)*	9.8 (1.4)	9.4 (1.5)	9.4 (1.2)	9.0 (1.3)
*Follicles on day 8 ≥11 mm, mean (SD)*	12.0 (6.1)	13.4 (6.4)	11.4 (5.7)	11.4 (5.8)
*Serum E*_*2*_*on day 8, pmol/l, median (P5, P95)*	2562	3285	2743	3274
	(837, 7524)	(840, 8918)	(987, 7487)	(1068, 8184)
*Follicles on day of hCG ≥11 mm, mean (SD)*	15.6 (6.5)	16.1 (6.9)	14.1 (6.0)	13.3 (5.8)
*E*_*2*_*on day of hCG, pmol/l, median (P5, P95)*	4239	4899	4110	4808
	(1512, 11,891)	(1762, 11,671)	(1501, 9762)	(1740, 10,129)
*No. of oocytes retrieved, mean (SD)*	14.1 (8.1)	14.0 (7.9)	12.8 (6.6)	12.4 (6.5)
*No. of embryos obtained on day 3, mean (SD)*	8.2 (5.6)	8.3 (5.4)	7.5 (4.8)	7.4 (4.6)
*No. of GQEs obtained on day 3, mean (SD)*	4.5 (4.2)	4.6 (4.4)	4.4 (3.8)	4.4 (3.8)
*No. of GQEs transferred, mean (SD)*	1.2 (0.8)	1.3 (0.8)	1.3 (0.8)	1.4 (0.8)
*Cycle cancellation rate, n (%)*	24 (7.0)	34 (9.2)	19 (5.4)	17 (4.7)

*SD* standard deviation, *P5* 5th percentile, *P95* 95th percentile, *E*_*2*_ estradiol, *GQEs* good-quality embryos.

**Table 2:** Estimated effect in percentage points (Δ) and associated 95% confidence interval (95% CI) of the covariates included in Model 1 and Model 2 on ongoing pregnancy rate

**Factors included in model**	**Categories**	Model 1*	**Model 2**^**†**^
		**Δ (95% CI)**	**Δ (95% CI)**
*Start treatment*	Day 2 versus day 3	-4.3 (-9.4, 0.8)	
*hCG delay*	No delay versus 1-day delay	1.8 (-4.1, 7.6)	
*rFSH on day of hCG*	Yes versus no	3.2 (-2.1, 8.6)	
*rFSH from day 8*	Step-down versus fixed dose		-5.8 (-13.0, 1.4)
*Treatment group*	Corifollitropin alfa versus rFSH	1.1 (-4.0, 6.2)	1.1 (-4.8, 6.9)
*Age*	Per year increase	-1.1 (-1.9, -0.3)	-0.9 (-1.8, 0.0)
*Region*	North America versus Europe	15.5 (10.2, 20.7)	12.6 (6.8, 18.5)

*Model 1 is based on Analysis Set 1, i.e., all patients who received *hCG* and for whom no rFSH dose was withheld for one or more consecutive days including the day of *hCG*.

^†^Model 2 is based on Analysis Set 2, i.e., all patients who received *hCG* on or after day 9 and for whom no *rFSH* dose was withheld for one or more consecutive days from day 8 including the day of *hCG*.

#### hCG administered on the day the hCG criterion was reached or 1 day later

In both the corifollitropin alfa arm and the rFSH arm, the number of oocytes retrieved was similar for patients who received hCG when the criterion was reached and for patients with a 1-day delay in hCG administration (Table 3). Cycle cancellation rates were higher in the corifollitropin alfa arm compared with the rFSH arm in patients who received hCG on the day the hCG criterion was met. The ongoing pregnancy rates for patients with hCG administration on the day the criterion was reached and for those with a 1-day delay were 40.7% and 39.4% in the corifollitropin alfa group and 37.8% and 42.9% in the rFSH group, respectively (Figure 1). The estimated difference (95% CI) in ongoing pregnancy rate between patients who received hCG when the criterion was reached and those with a 1-day delay was 1.8% (-4.1%, 7.6%) when adjusted for region, treatment group, age, and treatment flexibility options (i) and (iii) (Table 2). The estimated difference ratio was -2.0% (-7.8%, 3.8%) when adjusted for treatment group only and 2.0% (-3.8%, 7.8%) when adjusted for treatment group, age, and region (data not shown).

**Table 3:** Ovarian response and clinical outcomes: received hCG as soon as criterion was reached or 1-day delay (Analysis Set 1)

	**Corifollitropin alfa**		**rFSH**	
	**No delay**	**Delay**	**No delay**	**Delay**
	**n = 492**	**n = 188**	**n = 516**	**n = 191**
*Duration of stimulation, days, mean (SD)*	9.4 (1.4)	9.9 (1.3)	9.1 (1.3)	9.5 (1.0)
*Follicles on day 8 ≥11 mm, mean (SD)*	12.8 (6.6)	12.8 (5.5)	11.4 (5.9)	11.5 (5.1)
*Serum E*_*2*_*on day 8, pmol/l, median (P5, P95)*	3019	2753	2965	3097
	(837, 8588)	(1013, 7634)	(1017, 8184)	(1046, 7450)
*Follicles on day of hCG ≥11 mm, mean (SD)*	16.0 (6.9)	15.6 (5.5)	13.8 (6.2)	13.6 (5.0)
*E*_*2*_*on day of hCG, pmol/l, median (P5, P95)*	4551	4881	4477	4331
	(1629, 11,450)	(1883, 11,964)	(1718, 9872)	(1758, 10,349)
*No. of oocytes retrieved, mean (SD)*	14.2 (8.3)	14.1 (6.7)	12.4 (6.6)	13.3 (6.3)
*No. of embryos obtained on day 3, mean (SD)*	8.1 (5.7)	8.9 (4.9)	7.4 (4.7)	7.8 (4.5)
*No. of GQEs obtained on day 3, mean (SD)*	4.4 (4.3)	5.4 (4.2)	4.3 (3.7)	4.8 (3.9)
*No. of GQEs transferred, mean (SD)*	1.2 (0.8)	1.4 (0.7)	1.3 (0.8)	1.3 (0.8)
*Cycle cancellation rate, n (%)*	45 (9.1)	10 (5.3)	23 (4.5)	10 (5.2)

*SD* standard deviation, *P5* 5th percentile, *P95* 95th percentile, *E*_*2*_ estradiol, *GQEs* good-quality embryos.

#### rFSH on the day of hCG administration or not

Patients who received rFSH on the day of hCG compared with those who did not had a slightly higher number of oocytes retrieved in the corifollitropin arm and a similar number of oocytes retrieved in the rFSH arm (Table 4). Cycle cancellation rates were higher in the corifollitropin alfa arm than the rFSH arm. The ongoing pregnancy rates for patients who received rFSH on the day of hCG and for those who did not were 43.1% and 36.4% in the corifollitropin alfa arm and 39.7% and 37.6% in the rFSH arm, respectively (Figure 1). The estimated difference (95% CI) in ongoing pregnancy rate between patients receiving rFSH on day of hCG and those who did not was 3.2% (-2.1%, 8.6%) when adjusted for region, treatment group, age, and treatment flexibility options (i) and (ii) (Table 2). The estimated difference ratio was 4.3% (-1.0%, 9.6%) when adjusted for treatment group only and 3.0% (-2.2%, 8.2%) when adjusted for treatment group, age, and region (data not shown). There were no substantial differences among subgroups with respect to the incidence of OHSS (data not shown).

**Table 4:** Ovarian response and clinical outcomes: received rFSH on day of hCG or did not (Analysis Set 1)

	**Corifollitropin alfa**		**rFSH**	
	**No**	**Yes**	**No**	**Yes**
	**n = 247**	**n = 469**	**n = 255**	**n = 466**
*Duration of stimulation, days, mean (SD)*	9.6 (1.3)	9.6 (1.5)	9.3 (1.3)	9.1 (1.2)
*Follicles on day 8 ≥11 mm, mean (SD)*	13.4 (6.0)	12.3 (6.5)	11.1 (5.1)	11.5 (6.0)
*Serum E*_*2*_*on day 8, pmol/l, median (P5, P95)*	3382	2697	3075	2938
	(1039, 8441)	(793, 8147)	(1057, 9432)	(995, 7854)
*Follicles on day of hCG ≥11 mm, mean (SD)*	16.7 (6.5)	15.3 (6.7)	13.6 (5.7)	13.8 (6.1)
*E*_*2*_*on day of hCG, pmol/l, median (P5, P95)*	5468	4221	4514	4331
	(1982, 12,038)	(1497, 11,120)	(1736, 10,349)	(1600, 9799)
*No. of oocytes retrieved, mean (SD)*	14.8 (8.1)	13.6 (7.8)	12.4 (6.2)	12.7 (6.7)
*No. of embryos obtained on day 3, mean (SD)*	8.5 (5.4)	8.1 (5.5)	7.3 (4.4)	7.5 (4.8)
*No. of GQEs obtained on day 3, mean (SD)*	4.7 (4.1)	4.5 (4.4)	4.4 (3.5)	4.4 (3.9)
*No. of GQEs transferred, mean (SD)*	1.2 (0.8)	1.3 (0.8)	1.3 (0.8)	1.3 (0.7)
*Cycle cancellation rate, n (%)*	22 (8.9)	36 (7.7)	12 (4.7)	24 (5.2)

*SD* standard deviation, *P5* 5th percentile, *P95* 95th percentile, *E*_*2*_ estradiol, *GQEs* good-quality embryos.

#### Step-down or fixed-dose rFSH

In both the corifollitropin alfa and rFSH treatment groups, patients who received a reduced (step-down) rFSH dose (<200 IU) from day 8 had numerically higher estradiol levels on day 8 and on the day of hCG, more follicles ≥11 mm on the day of hCG, and more oocytes retrieved than patients who received a fixed-dose of rFSH (200 IU) from day 8 (Table 5). Cycle cancellation rates were higher in the corifollitropin alfa arm than the rFSH arm. The ongoing pregnancy rates for patients who received rFSH step-down or fixed-dose rFSH from day 8 were 37.0% and 40.6% in the corifollitropin alfa arm and 31.6% and 40.7% in the rFSH arm, respectively (Figure 1). The estimated difference (95% CI) in ongoing pregnancy rate between step-down and fixed-dose was -5.8% (-13.0%, 1.4%) when adjusted for treatment group, age, and region (Table 2).

**Table 5:** Ovarian response and clinical outcomes: step-down or fixed dose rFSH from day 8 (Analysis Set 2)

	**Corifollitropin alfa**		**rFSH**	
	**Step-down**	**Fixed dose**	**Step-down**	**Fixed dose**
	**n = 138**	**n = 409**	**n = 79**	**n = 430**
*Duration of stimulation, days, mean (SD)*	10.0 (1.1)	10.1 (1.3)	9.8 (1.1)	9.8 (1.0)
*Follicles on day 8 ≥11 mm, mean (SD)*	15.2 (5.1)	10.5 (5.4)	14.1 (5.3)	9.6 (4.7)
*E*_*2*_*on day 8, pmol/l, median (P5, P95)*	4734	2341	4184	2415
	(1431, 8918)	(738, 6019)	(995, 10,606)	(910, 6276)
*Follicles on day of hCG ≥11 mm, mean (SD)*	20.5 (5.3)	14.1 (6.2)	18.0 (6.1)	12.6 (5.3)
*Serum E*_*2*_*on day of hCG, pmol/l, median (P5, P95)*	7817	4147	7212	4221
	(2393, 13,799)	(1497, 10,239)	(1655, 16,772)	(1696, 9726)
*No. of oocytes retrieved, mean (SD)*	19.4 (8.1)	12.4 (7.4)	16.5 (6.4)	11.8 (6.4)
*No. of embryos obtained on day 3, mean (SD)*	11.8 (6.0)	7.5 (5.2)	10.2 (5.2)	7.1 (4.5)
*No. of GQEs obtained on day 3, mean (SD)*	6.5 (5.7)	4.1 (3.8)	5.5 (4.4)	4.3 (3.7)
*No. of GQEs transferred, mean (SD)*	1.1 (0.8)	1.3 (0.8)	1.3 (0.7)	1.3 (0.8)
*Cycle cancellation rate, n (%)*	15 (10.9)	30 (7.3)	4 (5.1)	23 (5.3)

*SD* standard deviation, *P5* 5th percentile, *P95* 95th percentile, *E*_*2*_ estradiol, *GQEs* good-quality embryos.

#### Corifollitropin alfa or rFSH treatment regimen

The ongoing pregnancy rate was similar in the two treatment groups: the estimated treatment effect (corifollitropin alfa - rFSH) was around 1% in both models (Table 2). For each of the four treatment flexibility options, its interaction with treatment group was tested and appeared to be not significant (in all cases *P* > 0.15, data not shown).

## Discussion

### Key results

The results of this *post hoc* analysis show that reducing the daily dose of rFSH from stimulation day 8 onward in a corifollitropin alfa or rFSH treatment regimen offers the flexibility to individualize care, and delaying the day of hCG administration does not compromise ongoing pregnancy rates. Treatment flexibility when starting ovarian stimulation was shown to have no statistically significant effect on clinical outcome in patients treated with corifollitropin alfa or rFSH in a GnRH antagonist protocol, but the pregnancy rate was estimated to be 4.3% lower in patients starting treatment on menstrual cycle day 2 and 5.8% lower in patients who received rFSH step-down. Starting stimulation too early could disturb complete shedding of the endometrium due to an early rise in estradiol and, thus, impair receptivity [7].

### Interpretation

The findings of this *post hoc* analysis are supported by those of several prior studies on treatment flexibility. With regard to a step-down or fixed dose of FSH, a meta-analysis of 10 studies (1952 IVF cycles) comparing a daily dose of 100 IU versus 200 IU rFSH and 150 IU versus 200 IU rFSH or higher showed that although the oocyte yield was greater in the >200 IU/day dose group, the pregnancy rates were similar when compared with the lower dose groups. The risk of an insufficient response to ovarian stimulation was greatest in the 100 IU/day dose group and the risk of developing OHSS was greater in the >200 IU/day dose group [8]. The authors concluded that the optimal daily rFSH stimulation dose is 150 IU in presumed normal responders aged <39 years undergoing IVF. A step-down approach for ovulation induction was also deemed more appropriate to avoid multiple follicular development in women with polycystic ovarian syndrome [9]. In addition, a step-down rFSH approach was shown to improve implantation rates and pregnancy rates in high responders [10]. Patients in the corifollitropin alfa or rFSH arms who received a step-down approach, in this *post hoc* analysis, had higher estradiol levels on day 8 and on the day of hCG, more follicles ≥11 mm on the day of hCG, and more oocytes retrieved than patients who received a fixed dose. However, the decision of a step-down or fixed-dose rFSH regimen was at the discretion of the investigator and rFSH was generally reduced in patients who had a high ovarian response. This process reflects clinical practice.

Administration of hCG for ovulation induction within 24 h after termination of rFSH has also been associated with good outcomes in patients undergoing COS in previous studies [11,12]. Delaying hCG administration has been shown to result in a higher incidence of endometrial advancement on the day of oocyte retrieval in donors stimulated with rFSH and GnRH antagonists [13]. Additionally, a large randomized controlled trial evaluating the effect of prolonging the follicular phase by delaying hCG administration on IVF outcome showed that oocyte or embryo quality was not affected in patients who received hCG as soon as the criterion was met or 2 days later [14]; however, delaying hCG administration was associated with a significantly lower ongoing pregnancy rate. Similar pregnancy rates have been reported with administration of hCG as soon as there were three or more follicles ≥16 mm or the day after [15]. In this *post hoc* analysis, it was shown that administration of hCG on the day hCG criterion was reached or 1 day later did not significantly affect ongoing pregnancy rates in patients treated with corifollitropin alfa or rFSH. However, in the current study population more subjects with hCG delay were from IVF centers in North America where higher ongoing pregnancy rates, compared with Europe, have previously been reported [6]. This has skewed the data in favor of ongoing pregnancy in the hCG delay population as described in the Results section: the estimated ongoing pregnancy rate difference between no delay and 1-day delay increased from -2.0% when only adjusted for treatment group to +2.0% when adjusted for factors age and region as well. Administration of rFSH on the day of hCG or no rFSH on the day of hCG also did not affect the ongoing pregnancy rates in the corifollitropin alfa or rFSH arm.

Treatment flexibility on the starting day of stimulation is often applied to avoid weekend oocyte retrievals. Comparison of clinical outcomes has been previously assessed in patients starting stimulation on menstrual cycle day 2 or menstrual cycle day 5 [16]. Starting stimulation on cycle day 5 resulted in more cancellations due to insufficient response and a lower percentage of oocytes retrieved than patients starting stimulation on cycle day 2. There was no difference in the ongoing pregnancy rates between the two groups. However, a higher ongoing pregnancy rate was observed in patients starting stimulation on cycle day 3, as reported in the primary manuscript of the Engage trial [3] and in a recent retrospective analysis of patients treated with rFSH in the Engage trial [17]. In this analysis of the Engage trial, in which the probability of pregnancy was adjusted for factors treatment group, region, age, and treatment flexibility options (ii) (no hCG delay versus 1-day delay) and (iii) (rFSH administered on day of hCG or not), the estimated day 2 minus day 3 ongoing pregnancy rate (95% CI) was -4.3% (-9.4%, 0.8%). The estimated pregnancy rate for starting day 2 is numerically lower than for starting day 3; however, as the 95% CI includes zero we cannot conclude that there is any difference.

A recent systematic review suggested that there may be an increased risk of OHSS following treatment with corifollitropin alfa [18]. Errors in that review have already been pointed out by Mannaerts et al. [19]. Nonetheless, a numerically higher risk (although not-statistically significant) remains which is linked to the degree of ovarian response. However, we note that it is becoming clear that with appropriate use of a GnRH agonist trigger in women at risk of over-response, OHSS can be largely eliminated [20].

This *post hoc* analysis provides valuable insight on the impact of “real-life” treatment flexibility on clinical outcomes in patients treated with corifollitropin alfa or rFSH.

### Limitations

As with any *post hoc* analysis, conclusions drawn from the analyses in this report must be viewed with caution because all of the comparisons were *post hoc* and should be confirmed by prospective studies.

## Conclusions

Treatment flexibility at the start or completion of ovarian stimulation does not substantially affect clinical outcome in patients treated with corifollitropin alfa or in those treated with daily rFSH for the first 7 days of COS using a GnRH antagonist protocol.

## Competing interests

K. Gordon and H. Witjes are employees of Merck Inc. A. Leader and P. Devroey have no potential competing interests.

## Authors’ contributions

AL and PD interpreted the data and revised the paper critically for important intellectual content. KG interpreted the data and drafted the paper. HW analyzed and interpreted the data and drafted the manuscript. All authors gave final approval of the version to be published.
